# Questionnaire-Based Assessment of Patients’ Knowledge, Attitude, and Practices Regarding Restorative and Endodontic Treatment at a Multi-disciplinary Tertiary Care Hospital of Jharkhand, India

**DOI:** 10.7759/cureus.59526

**Published:** 2024-05-02

**Authors:** Sumit Mohan, Butta Viswanath, Gaurav Kumar, Harsh Priyank

**Affiliations:** 1 Department of Conservative, Endodontics & Aesthetic Dentistry, Dental Institute, Rajendra Institute of Medical Sciences, Ranchi, IND

**Keywords:** filling, root canal treatment (rct), questionnaire, oral health, knowledge

## Abstract

Background: The majority of Indians living in smaller cities and villages don't know much about oral health and how to address it. Thus, this research seeks to assess the endodontic and restorative treatment knowledge, attitudes, behaviors, and perceptions of patients who visit the Dental Institute at the Rajendra Institute of Medical Sciences in Ranchi.

Methods: This study was conducted on 771 subjects over 2 months at the outpatient department (OPD) of the dental institute, using a prefabricated questionnaire. The participants were divided into three groups based on age. A modified questionnaire consisting of 20 questions obtained from previous studies was provided to the subjects. The first part of the questionnaire was related to demographic details while the second part comprised questions regarding the knowledge of the participants. The third part emphasized on attitude aspect while the last part comprised practice questions.

Results: It was observed that 682 (85%) of the participants had prior information about root canal treatment (RCT) and filling and 555 (72%) thought it to be an alternative to extraction. While 528 (68.5%) participants stated about undergoing RCT, 679 (88%) subjects propagated their recommendation to family and friends. Five hundred thirteen (66%) subjects highlighted anxiety during anesthetic administration.

Conclusion: With increasing awareness and information, traditional extraction has given way to the recognition that RCT and filling can salvage a tooth. Patient acceptance of RCT and filling as treatment alternatives may be enhanced by healthcare education and mass activities.

## Introduction

The WHO reports dental caries as the most common chronic disease which requires prompt management because if left untreated, it might lead to pain resulting in tooth loss thereby affecting a person’s quality of life [1-5]. Factors like function and aesthetics motivate the patient to seek dental treatment [6]. Root canal treatment (RCT) and restorative therapy (Filling) are restitutive dental procedures involving direct interactions between the dentist and the patient where the dentist takes into consideration factors like clinical criteria, the patient's socioeconomic status, and the patient’s expectations to modify of behaviors of patients in a positive way which might help patients accept the procedures [7-10].

Health services provide interventions at primary, secondary, and tertiary levels. So, the study aimed to assess the level of knowledge, attitude, and practice among patients at the Dental Institute, Rajendra Institute of Medical Sciences, a tertiary care facility in Jharkhand, regarding RCT and fillings. By gaining insights into patients' perspectives and behaviors, the institute aims to provide its services and educational programs to better meet patients' needs and preferences.

The study seeks to explore the factors that influence patients' perceptions and how their knowledge and attitudes impact decision-making regarding dental treatment, including adherence to recommended procedures. Furthermore, the research aims to identify potential barriers to patient acceptance and compliance with RCT and fillings, paving the way for strategies to overcome these obstacles. Ultimately, the study aims to inform the institute on ways to enhance patient education and communication, thus improving treatment acceptance and outcomes.

## Materials and methods

Study location and ethics approval

This study was conducted at the dental outpatient department (OPD), where adult patients who volunteered to participate were enrolled over 2 months. Ethical clearance was obtained from the Institutional Ethical Committee, referenced as letter no 13 dated 15/4/2020, with the institutional review board number (IRB) IEC/ECR/769/INST/JH/2015/RR-18. The study strictly adhered to ethical guidelines. Exclusion criteria included individuals who were uninterested, unwilling, edentulous, minors, those with impaired medical conditions, or those requiring emergency care. Written consent was obtained from all participants.

Inclusion and exclusion criteria

Adult patients attending the dental OPD who voluntarily consented to participate were included in the study. Exclusion criteria comprised individuals who were uninterested or unwilling to participate, edentulous patients, minors, individuals with impaired medical conditions that could affect their ability to respond accurately to the questionnaire, and those requiring immediate emergency dental care. All included participants provided written informed consent before their involvement in the study.

Sampling technique

Convenience sampling was employed, and 771 (95%) out of 811 eligible patients consented to participate during the stipulated study period. Participants were categorized into three groups based on age for comparative analysis. The sample size was determined using the formula, n= N/1+N(e2)

Where: n = sample size, N = total population size, e = margin of error

Data collection

A modified questionnaire, comprising 20 questions derived from previous studies [1-3,7,10-15], was administered to the participants. The questionnaire was divided into four parts: the first part gathered demographic details, the second part assessed participants' knowledge, the third part evaluated their attitudes, and the final part addressed their practices regarding the subject under investigation. This comprehensive questionnaire ensured a thorough assessment of participants' perspectives and behaviors related to the study objectives. The reliability of the questionnaire was done, through internal consistency analysis (such as Cronbach's alpha), which enhanced the methodological transparency of the study.

Statistical analysis

Data collected from the questionnaires were entered into a statistical software program for analysis. Descriptive statistics such as percentages were calculated to summarize the demographic characteristics of the participants and their responses to the questionnaire items. Inferential statistical tests, such as the chi-square test or analysis of variance (ANOVA), were employed to assess the associations between demographic variables and participants' knowledge, attitudes, and practices. Intergroup comparison was assessed through the ANOVA test. A p-value of less than 0.05 was considered statistically significant. Additionally, subgroup analyses were conducted based on age groups to explore potential differences in responses. This rigorous statistical analysis provided valuable insights into the relationships between various factors and the outcomes of interest, contributing to the robustness and reliability of the study findings.

## Results

As shown in Table 1, most of the study participants were females, whose distribution by age included those less than 30 years old, those aged between 30 and 60 years old, and those above 60 years old. All three age distributions had the highest frequency and least participation in the order listed; for example, the fewest male participants were recorded in the category of those aged below 30 years, and its frequencies were 14, 2, and 7. Furthermore, the distribution was categorized according to the participants’ level of education, such as illiterate, primary, secondary, graduate, or postgraduate levels, and the number of participants. The table provided a figure value for the chi-square of each variable and the p-value to determine if age and investigation were dependent on gender (Table 1).

**Table 1:** Demographic characteristics of the study participants

Variables		Total	Males	Females	P-value	
Age	Less than 30 years	302 (39.16%)	138 (45.69%)	164 (54.31%)	Chi-square = 4.38	P-value = 0.112
	From 30 – 60 years	408 (52.9%)	176 (43.13%)	232 (56.87%)		
	Above 60 years	61 (7.9%)	35 (57.37%)	26 (42.63%)		
Education Status	Illiterate	61 (7.9%)	38	23	Chi-square = 23.79	P-value = 0.001
	Primary	138 (17.89%)	67	71		
	Secondary	363 (47.1%)	174	189		
	Graduate	177 (23.0%)	64	113		
	Postgraduate	32 (4.1%)	6	26		

Overall, by recognizing the implications of demographic trends on dental care utilization and treatment outcomes, clinicians and public health practitioners can implement targeted strategies to promote oral health equity and improve the delivery of dental services to diverse populations.

Most of the participants’ responses to statements on the panels are presented in this table. The responses regarding dental care included a statement of whether RCT was superior to tooth extraction, whether a filling was always enough to save a tooth, whether after RCT, teeth become weak, and whether a crown is always placed on teeth after an RCT. Although some responses matched, others had differences due to a lack of knowledge or preconceived notions about dental treatment. The chi-square value was calculated from the results of the p-value (Table 2).

**Table 2:** Response of the participants on knowledge statements

Questions		Total	Males	Females	Chi-square value	P-value
Is RCT better than extraction?	Yes	72%	238	317	4.54	0.033
	No	28%	111	105		
Does filling prevent RCT/extraction?	Yes	72.2%	252	303	0.01	0.901
	No	27.8%	97	119		
Does filling weaken the tooth?	Yes	49.5%	146	236	15.17	<0.000
	No	50.5%	203	186		
RCT results in tooth becoming dead?	Yes	56%	169	263	14.97	<0.000
	No	44%	180	159		
After RCT the tooth should be given a crown?	Yes	61.9%	184	293	22.61	<0.000
	No	37.8%	165	129		
Correct knowledge (mean + SD)	3.16+1.590					

The participant’s knowledge about endodontic and restorative therapy where 84.5% of participants had heard about the treatments of which 61% had heard about it from family and friends, 10% from books and newspapers, 14% from doctors while 15% got to know about it through the internet (Table 3).

**Table 3:** Source of information about root canal treatment and filling

Source of Information	Percentage (%)
Family, friends	61%
Internet	15%
Doctors, dentists	14%
Print- Newspaper, books, journals	10%

The data presented highlights the attitudes and perceptions of patients toward RCT and fillings post-therapy. The majority of participants, comprising 88.5%, identified multiple appointments as the most significant drawback of RCT. This finding underscores the inconvenience associated with RCT, likely due to the need for multiple visits to complete the treatment process, which can disrupt daily schedules and require additional time commitments from patients. Additionally, a notable proportion of participants, accounting for 31.5%, expressed discomfort with the use of anesthetic injections during dental procedures. This sentiment reflects the apprehension and anxiety commonly associated with injections, which may contribute to patient reluctance or apprehension toward undergoing RCT. However, it is reassuring to note that only a small fraction, 3.5% of subjects, reported drilling as being painful, indicating that the discomfort experienced during RCT procedures is relatively minimal for the majority of patients.

Furthermore, a considerable portion of participants, constituting 28.5%, found radiographs to be annoying. This perception may stem from the discomfort caused by the positioning of the imaging equipment and the need to hold still for extended periods during radiographic examinations. Despite these concerns and inconveniences associated with RCT, an overwhelming majority of patients, comprising 88% of the sample, ultimately concluded that RCT and restoration were preferable options to tooth extraction. This finding suggests that despite the perceived drawbacks and discomfort associated with RCT procedures, patients value the preservation of their natural teeth and recognize the long-term benefits of undergoing RCT in terms of maintaining oral health and function. Overall, the data elucidates the complex interplay between patient perceptions, treatment preferences, and the perceived benefits and drawbacks of dental procedures, underscoring the importance of patient education and communication in facilitating informed decision-making and enhancing patient satisfaction with dental care (Table 4).

**Table 4:** Response of the participants on attitudes statements

Questions	Responses	Percentages	Males	Females	Chi-square value	P-value
What is the biggest disadvantage of RCT?	Interappointment pain	4.5%	21	14	3.8511	0.278
	Multiple appointments	88.5%	306	376		
	None	5%	15	24		
	I know	2%	7	8		
Did you mind getting an anesthetic injection?	No	66.5%	194	319	36.93	<0.000
	Yes	31.5%	149	94		
	I know	2%	6	9		
Did you mind drilling on your tooth?	No	95.5%	334	402	0.206	0.902
	Yes	3.5%	12	15		
	I know	1%	3	5		
Did you mind going for multiple X-rays on your tooth?	No	70.5%	250	294	1.014	0.602
	Yes	28.5%	97	123		
	I don’t know	1%	2	5		
Would you recommend RCT or Filling to your family or friends?	No	9.5%	32	41	0.153	0.926
	Yes	88%	309	370		
	I don’t know	2.5%	8	11		
Correct Attitude (mean + SD)	4.32+1.253					

Table 5 presents the participants' practices and behaviors related to dental treatment choices and adherence to dental recommendations. The questions cover topics such as compliance with dentist recommendations for RCT, preferences for RCT over extraction, willingness to undergo additional dental procedures like pulp capping, and treatment choices for anterior tooth trauma. The table provides the percentage of participants who responded to each question, categorized by gender. Additionally, chi-square test statistics and p-values are provided to assess the significance of any gender differences in practice responses (Table 5).

**Table 5:** Response of the participants on practice statements

Questions	Responses	Percentages	Males	Females	Chi-square value	P-value
What do you do if your dentist advises you RCT?	Obey him	68.5%	238	290	14.7099	0.002
	Want to go for extraction of tooth	19.5%	56	94		
	Use medication to delay treatment	8%	41	21		
	I don’t know	4%	14	17		
After knowing about RCT does it affect your treatment choice?	Treatment remains RCT	72%	248	307	0.2969	0.862023
	Treatment is extraction	22%	79	91		
	I don’t know	6%	22	24		
Will you go for a pulp capping procedure to avoid RCT?	Yes	95%	326	406	5.211	0.738
	No	3.5%	18	9		
	I don’t know	1.5%	5	7		
In case of anterior tooth trauma, your treatment of choice would be…	RCT+ filling	75.5%	262	320	1.397	0.497
	Extraction	21.5%	79	87		
	I don’t know	3%	8	15		
If you have choice between RCT and extraction, you would choose…	RCT	71%	254	293	1.2165	0.544
	Extraction	23.5%	78	103		
	I don’t know	5.5%	17	26		
Correct Practice (mean + SD)	5.56+0.907					

Table 6 displays the correlation coefficients that measure the relationship between the participants' knowledge (K), attitude (A), and practice (P) regarding dental treatments. Correlation coefficients provide a measure of the intensity and orientation of the linear connection between two variables. A positive correlation indicates that when one variable grows, the other variable likewise tends to increase, whereas a negative correlation indicates that as one variable increases, the other variable tends to decline. The table also includes p-values to evaluate the statistical significance of the observed associations.

**Table 6:** Correlation between KAP KAP: knowledge, attitude, and practice

Variable	Knowledge		Attitude		Practice
Knowledge	-	-	-	-	-
Attitude	r value = 0.535	p-value = 0.001	-	-	-
Practice	r value = 0.481	p-value = 0.001	r value = 0.243	p-value = 0.001	-

## Discussion

This study focused on a specific group of people. Out of the 771 participants, 54.74% were female, 52.9% were between the ages of 30 and 60, and only 61 participants (7.9%) were 60 years old or older. The duration of the evaluation session was 2 months. According to research done by Pradhan and colleagues, [11] while Iyer et al. found that 47.66% of the patients were females above the age of 18, [12] conducted a study on 508 subjects. Our study showed that 84.5% of the patients were aware of restorative and endodontic procedures, and a majority of the subjects had received some form of instruction on the topic. Research indicates that patients' level of knowledge and awareness significantly impact their decision-making process [13,14].

Seventy-three percent of the subjects believed in the timely management of caries to prevent RCT or extraction in the future making our findings significantly higher than other studies [11,15]. A total of 61% of participants in this research reported hearing about RCTs and fillings from people they knew. Results of other studies highlighted the role of audio-visual aids, newspapers, dentists, relatives, friends, and family as a source of information about endodontic and restorative treatments [4,11,15].

A good coronal seal is essential for a successful RCT [16]. Some study participants (61.9%) realized the importance of the crown after RCT which is in agreement with the preexisting literature [5,17]. Conflicting results were observed by Ray and Trope who reported that a good restoration was sufficient after endodontic treatment [18]. A patient’s choice of treatment is governed by factors like the doctor’s skill, convenience, and cost [19-21]. There was no cost of the treatment in the present study as it was conducted in a government institution. In the present study, patients believed that multiple appointments, drilling, intraoral radiographs, and anesthetic injections were the unpleasant experiences associated with endodontic and restorative treatments which were in agreement with observations of other researchers [22,23].

Dissimilarities in demographics, social and religious practices, educational attainment, and healthcare systems in the area may explain why certain studies have shown different outcomes.

The study limitations include that the study's findings may be limited by sample bias, as it was conducted at a single dental institute in Ranchi, potentially not fully representative of the broader population in smaller cities and villages. Moreover, relying on self-reported data from participants introduces the possibility of response bias, where participants may provide socially desirable answers or overstate their knowledge and behaviors. The questionnaire's limited scope may also constrain the depth of understanding, as it does not thoroughly explore socioeconomic status, cultural beliefs, or previous dental experiences. Additionally, the cross-sectional design precludes establishing causal relationships or assessing changes over time, while potential confounding variables such as previous dental history or socioeconomic status were not accounted for, potentially introducing bias into the results.

Future research could consider longitudinal study designs to track changes in patient perceptions over time and multi-center studies to capture diverse perspectives across various geographical regions and healthcare settings. Qualitative research methods, such as focus groups or in-depth interviews, could complement quantitative findings by providing deeper insights into the underlying reasons behind patient attitudes and behaviors. Furthermore, intervention studies evaluating the effectiveness of healthcare education interventions or mass awareness campaigns could inform the development of targeted strategies to improve oral health literacy and treatment outcomes among underserved populations. Addressing these limitations and exploring these avenues for future research would contribute to a more comprehensive understanding of patient perspectives on dental care and inform the development of effective interventions to address oral health disparities in India.

## Conclusions

The study outcomes offer a significant role in patients' understanding, attitudes, behaviors, and perceptions concerning endodontics and restorative treatments at the Dental Institute of the Rajendra Institute of Medical Sciences in Ranchi. It is encouraging to note that a significant majority of participants had prior information about RCT and filling, with many considering them as viable alternatives to tooth extraction. Moreover, a considerable proportion of participants expressed willingness to undergo RCT themselves and recommended it to their family and friends, indicating a positive perception of these treatments. However, the high prevalence of anxiety during anesthetic administration among participants highlights a potential barrier to treatment acceptance that warrants attention. These findings underscore the importance of targeted patient education initiatives aimed at alleviating anxiety and promoting an understanding of the benefits of endodontic and restorative treatments. By addressing these factors, dental healthcare providers can work toward improving oral health outcomes and increasing treatment acceptance among patients in smaller cities and villages, ultimately contributing to better overall oral health in India.

## References

[1] Kumar H, Behura SS, Ramachandra S, Nishat R, Dash KC, Mohiddin G (2017). Oral health knowledge, attitude, and practices among dental and medical students in Eastern India - a comparative study. J Int Soc Prev Community Dent.

[2] Porhashemi S, Mahmodian J (1993). Evolutions of dental caries prevalence and prevention in Iran and other countries. J Dent Med Tehran Univ Med Sci.

[3] Samorodnitzky-Naveh GR, Geiger SB, Levin L (2007). Patients' satisfaction with dental esthetics. J Am Dent Assoc.

[4] Parirokh M, Zarifian A, Ghoddusi J (2015). Choice of treatment plan based on root canal therapy versus extraction and implant: A mini review. Iran Endod J.

[5] Sorensen JA, Martinoff JT (1985). Endodontically treated teeth as abutments. J Prosthet Dent.

[6] Tin-Oo MM, Saddki N, Hassan N (2011). Factors influencing patient satisfaction with dental appearance and treatments they desire to improve aesthetics. BMC Oral Health.

[7] Mirzadeh A, Gholami S, Rafiee F, Shahravan A (2013). An evaluation of the knowledge and attitude of school teachers regarding root canal therapy in Kerman, Iran. J Oral Health Oral Epidemiol.

[8] Erten H, Akarslan ZZ, Bodrumlu E (2006). Dental fear and anxiety levels of patients attending a dental clinic. Quintessence Int.

[9] Hajjaj FM, Salek MS, Basra MK, Finlay AY (2010). Non-clinical influences on clinical decision-making: A major challenge to evidence-based practice. J R Soc Med.

[10] Keogh T, Linden GJ (1991). Knowledge, attitudes and behaviour in relation to dental health of adults in Belfast, Northern Ireland. Community Dent Oral Epidemiol.

[11] Pradhan RJ, Shrestha R, Thappa A, Acharya BK (2019). Awareness and knowledge of root canal treatment: A survey. Int J Emerging Trends Sci Tech.

[12] Iyer AA, Nair R, Gupta P, Tavane NP, Parwar P (2018). Dental patients' knowledge and awareness and attitudes towards root canal treatment: A survey based research. Int J Res Sci Res.

[13] Doumani M, Habib A, Qaid N, Abdulrab S, Bashnakli AR, Arrojue R (2017). Patients’ awareness and knowledge of the root canal treatment in Saudi population: Survey-based research. Int J Dent Res.

[14] Janczarek M, Cieszko-Buk M, Bachanek T, Chałas R (2014). Survey-based research on patients’ knowledge about endodontic treatment. Pol J Public Health.

[15] Manasa N, Naik KB, Singh M, Moiz AA, Kudala VR, John NK (2023). Patient's knowledge and perception of endodontics treatment: An observational study. J Pharm Bioallied Sci.

[16] Goldstein RE (1993). Esthetic dentistry—a health service?. J Dent Res.

[17] Aquilino SA, Caplan DJ (2002). Relationship between crown placement and the survival of endodontically treated teeth. J Prosthet Dent.

[18] Ray HA, Trope M (1995). Periapical status of endodontically treated teeth in relation to the technical quality of the root filling and the coronal restoration. Int Endod J.

[19] Al-Hussyeen AJ (2010). Factors affecting utilization of dental health services and satisfaction among adolescent females in Riyadh City. Saudi Dent J.

[20] Moshkelgosha V, Mehrzadi M, Golkari A (2014). The public attitude towards selecting dental health centers. J Dent (Shiraz).

[21] Iqbal M, Jameel A, Girach MM, Murtaza Murtaza (2014). Factors affecting patient’s choice of dental services. Pak Oral Dent J.

[22] Priya S, Rathod D, Hazra S, Pathak L (2020). Evaluation and perspective towards endodontic treatment among patients visiting to a private dental college in Hazaribagh. Int J Appl Res.

[23] Mohan S, Thakur J (2018). Phobia of endodontic treatment: A myth or realty—a questionnaire based survey. Int J Adv Integ Med Sci.

